# Integrative analysis of FAERS, network toxicology, and Mendelian randomization identifies potential targets in paclitaxel-associated systemic sclerosis

**DOI:** 10.3389/fimmu.2026.1696820

**Published:** 2026-07-09

**Authors:** Xinfeng Wang, Chengyan Zhang

**Affiliations:** 1 Department of Pharmacy, Shanxi Province Cancer Hospital/Shanxi Hospital Affiliated to Cancer Hospital, Chinese Academy of Medical Sciences/Cancer Hospital Affiliated to Shanxi Medical University, Taiyuan, Shanxi, China; 2 Department of Gastroenterology, Shanxi Province Cancer Hospital/Shanxi Hospital Affiliated to Cancer Hospital, Chinese Academy of Medical Sciences/Cancer Hospital Affiliated to Shanxi Medical University, Taiyuan, Shanxi, China

**Keywords:** enrichment analysis, FAERS, Mendelian randomization, network toxicology, paclitaxel, protein–protein interaction network, systemic sclerosis

## Abstract

Systemic sclerosis (SSc) is a rare and complex autoimmune disease characterized by fibrosis of the skin and internal organs as well as vascular abnormalities. Studies have suggested that paclitaxel may induce adverse reactions resembling systemic sclerosis; however, the underlying mechanisms remain not fully understood. We retrieved reports of paclitaxel-associated SSc from the FDA Adverse Event Reporting System (FAERS). Potential shared targets between paclitaxel and SSc were identified through network toxicology analysis. Mendelian randomization (MR) was then used to explore associations between these targets and SSc susceptibility. Disproportionality analyses demonstrated significant safety signals linking paclitaxel with SSc, scleroderma, and scleroderma-like reactions. A total of 76 overlapping targets were identified between paclitaxel and SSc. Based on expression quantitative trait loci (eQTL) from the IEU OpenGWAS database, MR analysis suggested 11 targets potentially associated with SSc susceptibility. Functional enrichment analyses revealed that these genes were involved in oxidative stress response, regulation of cell death, lipid metabolism, and apoptosis. Among them, AKT1 and BCL2 were highlighted as central nodes in the protein–protein interaction network, representing candidate targets for further investigation. Molecular docking simulations provided exploratory computational evidence of potential interactions, which do not confirm functional or mechanistic roles. Overall, this study systematically explored potential molecular targets related to paclitaxel-associated SSc and provides hypothesis-generating insights that may guide future mechanistic studies and risk assessment strategies.

## Introduction

Systemic sclerosis (SSc) is a rare and complex autoimmune disease characterized by progressive fibrosis of the skin and internal organs, along with microvascular dysfunction and immune dysregulation (1, 2). Clinically, SSc commonly presents with skin thickening (scleroderma), Raynaud’s phenomenon—a vasospastic disorder affecting the fingers and toes—and involvement of internal organs such as the lungs, heart, and kidneys, which contributes to significant morbidity and mortality (3). Epidemiological studies estimate the annual incidence of SSc to be approximately 10–20 cases per million population, with a marked female predominance (female-to-male ratio of ~4:1) (4). While the exact etiology of SSc remains elusive, environmental triggers in genetically predisposed individuals are believed to play a significant role (5).

Increasing attention has been paid to drug-induced SSc, particularly in patients receiving specific chemotherapeutic or immunomodulatory agents (6, 7). Paclitaxel, a microtubule-stabilizing agent widely used in oncology, has been reported to induce scleroderma-like clinical manifestations in certain patients (8). Several case reports have documented cutaneous thickening and Raynaud’s phenomenon following paclitaxel treatment, while observational studies have further described systemic fibrosis closely resembling SSc (9, 10). However, the molecular mechanisms underlying these adverse events remain largely unexplored. Given that drug-induced SSc may be at least partially reversible, elucidating its pathogenesis is crucial for improving clinical risk prediction and guiding treatment strategies.

The FDA Adverse Event Reporting System (FAERS) is one of the world’s largest pharmacovigilance databases, collecting and monitoring spontaneous reports of drug-related adverse events (11, 12). FAERS has been widely used to identify potential drug safety signals, assess rare but serious adverse effects, and generate hypotheses for mechanistic studies (13, 14). Recent studies have also integrated FAERS data with systems biology and bioinformatics tools to explore drug-related toxicity pathways (15).

In this study, we utilized FAERS to identify safety signals of paclitaxel-associated SSc, then applied network toxicology to uncover potential shared molecular targets between paclitaxel and SSc. To verify the associations between paclitaxel and adverse reactions (AE) of SSc. We employed Mendelian randomization (MR), a powerful approach that uses genetic variants as instrumental variables to infer associations between exposures (gene expression) and outcomes (disease) (16). By integrating pharmacovigilance, network biology, and genetic epidemiology, this work aims to explore molecular associations related to paclitaxel-associated systemic sclerosis adverse events and prioritize candidate targets for future investigation.

## Materials and methods

### FAERS-based pharmacovigilance analysis

We extracted AE reports related to paclitaxel from the FDA Adverse Event Reporting System (FAERS, https://fis.fda.gov/extensions/FPD-QDE-FAERS/FPD-QDE-FAERS.html), covering the period from Q1–2004 to Q4 2024. Paclitaxel was approved in December 1992 (17); therefore, only reports from 2004 onward were included to ensure consistency in data collection.

Preferred terms (PTs) related to SSc were selected based on MedDRA terminology (18), including: SYSTEMIC SCLERODERMA, SCLERODERMA, SCLERODERMA-LIKE REACTION, SCLERODERMA OVERLAP SYNDROME, and CREST SYNDROME. Drug names were standardized using the WHO Drug Dictionary (version September 2024).

We analyzed key patient characteristics, including sex, age (categorized as <18, 18-44, 45-64, ≥65), weight (<80 kg or ≥80 kg), reporter occupation (e.g., consumer, pharmacist, physician), geographic region, and clinical outcome (e.g., hospitalization, disability, death). Outliers were excluded if age < 0 or > 120 years, or if weight > 400 kg (19). Given that a large proportion of reports had missing data (87.6%), body weight was included solely as a descriptive variable and was not used in formal disproportionality or subgroup analyses. FAERS data are derived from spontaneous reports and may be subject to underreporting or reporting bias; these analyses are therefore exploratory in nature.

**Figure 1:**
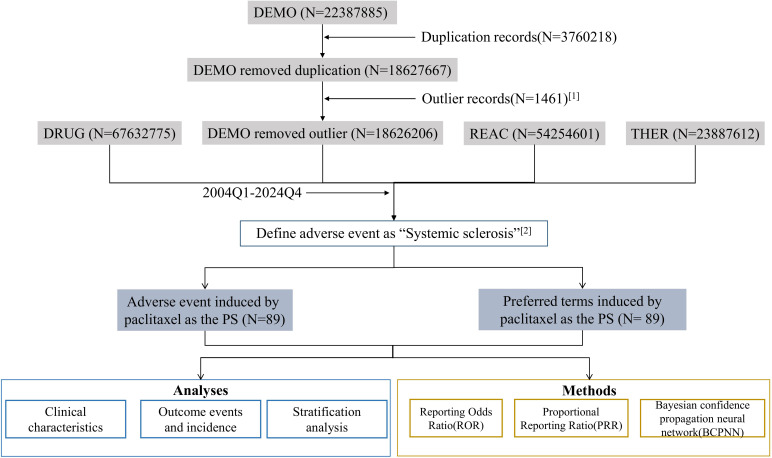
Schematic diagram illustrating the steps in collecting and analyzing adverse event reports related to paclitaxel-associated systemic sclerosis from the FAERS database (1). Records with age < 0 or > 120 years, and weight > 400 kg were considered outliers and excluded from analysis (2). The following preferred terms (PTs) were used to identify systemic sclerosis-related adverse events: SYSTEMIC SCLERODERMA, SCLERODERMA, SCLERODERMA-LIKE REACTION, SCLERODERMA OVERLAP SYNDROME, and CREST SYNDROME. DEMO: Contains patient demographic characteristics related to the adverse event reports. DRUG: Contains detailed information about the suspect and concomitant drugs involved in the report. REAC: Contains details about the adverse reactions experienced by the patient. THER: This table records information about the duration of a specific drug therapy, including the start and end dates of use. PS, primary suspect drug.

Disproportionality analysis was performed using standard signal detection metrics (20): reporting odds ratio (ROR), proportional reporting ratio (PRR), and Bayesian confidence propagation neural network (BCPNN). A signal was considered positive if it met predefined thresholds: ROR lower 95% CI > 1; PRR ≥ 2 with χ² ≥ 4; IC025 > 0. Signal strength was interpreted using ROR as the primary indicator, with PRR and BCPNN used for verification. Statistical analyses were performed using R version 4.4.1. The workflow of FAERS-based pharmacovigilance analysis is shown in **Figure 1**.

### Data sources

The chemical structure and SMILES string of paclitaxel were retrieved from the PubChem database (21). Potential drug targets were identified using CTD, STITCH, and SwissTargetPrediction databases, and standardized via UniProt (22, 23). SSc-related genes were obtained from GeneCards and DisGeNET using the keyword “systemic sclerosis” (24). Targets with a relevance score above the median were retained. Shared targets between paclitaxel and SSc were visualized using a Venn diagram.

Expression quantitative trait loci (eQTLs) for candidate genes were obtained from the IEU OpenGWAS Project (https://gwas.mrcieu.ac.uk/) (25). GWAS summary statistics for systemic sclerosis were obtained from the FinnGen R12 dataset (ID: finngen_R12_SYSTSCLE_STRICT) (26). All data were derived from European populations and were accessed on February 15, 2025.

### Mendelian randomization analysis

To satisfy core MR assumptions (relevance, independence, exclusivity, and cis-acting), SNPs were selected based on the following criteria: Strong association with gene expression (*P* < 5×10^-8^); Minor allele frequency (MAF) > 0.01; Linkage disequilibrium r² < 0.3 within ±100 kb; Located within ±500 kb of the gene’s transcription region; Palindromic SNPs and SNPs associated with potential confounders were excluded using the GWAS Catalog (27). Associations between target gene expression and SSc were estimated using five MR methods: inverse variance weighted (IVW, primary), MR-Egger, weighted median, weighted mode, and simple mode. False discovery rate (FDR) correction was applied with the following formula $FDR=\frac{Pvalue*Rank_{\max}}{P_{rank}}$. Heterogeneity was assessed using Cochran’s Q test and the I² statistic. A Q test *P* value < 0.05 was considered indicative of significant heterogeneity among SNPs. The I² statistic quantifies the proportion of total variation attributable to heterogeneity; an I² > 50% suggests moderate to substantial heterogeneity in the IVW estimates. Horizontal pleiotropy was evaluated using the MR-Egger intercept and the MR-PRESSO global test; *P* > 0.05 in both tests was interpreted as the absence of directional pleiotropy. Leave-one-out analysis was used to evaluate the influence of individual SNPs. Analyses were performed using the TwoSampleMR package in R 4.1.0, with α = 0.05.

### Functional enrichment analysis

Gene Ontology (GO) enrichment (biological process, cellular component, molecular function) and Kyoto Encyclopedia of Genes and Genomes (KEGG) pathway analyses were conducted on the 11 core candidate genes using the clusterProfiler R package (28). Significance was defined as adjusted P < 0.05. The top 10 GO and top 20 KEGG enriched terms were visualized.

### Protein–protein interaction network

The 11 candidate genes were uploaded to the STRING database to construct the PPI network. To provide a more comprehensive interaction context, first-order interactors of these genes were retrieved from the STRING database (version 11.5) with a minimum required interaction confidence score of 0.7. Network topology and centrality were analyzed using Cytoscape (v3.10.3). Hub genes were identified using the Maximal Clique Centrality (MCC) algorithm (29). These analyses are exploratory and intended to prioritize candidate genes for further investigation rather than confirm mechanistic roles.

### Molecular docking

Molecular docking was performed using CB-Dock2 (https://cadd.labshare.cn/cb-dock2/php/index.php) (30). Protein receptor structures were obtained from the Protein Data Bank (PDB IDs: 7NH4 for AKT1, 1G5M for BCL2) and prepared by removing crystallographic water molecules, assigning protonation states, and adding charges according to default CB-Dock2 protocols. Ligand structures were optimized using energy minimization before docking. Docking was conducted using the default scoring functions and parameters, and the pose with the lowest predicted binding energy was selected for analysis. Paclitaxel structure was obtained from PubChem (PubChem CID 36314). Docking affinity was assessed using AutoDock Vina scores. Binding energies below −5 kcal/mol were used as an exploratory screening criterion.

No reference ligands were included for benchmarking, and no molecular dynamics simulations were performed. Therefore, these docking results are considered exploratory and hypothesis-generating, intended to support candidate target prioritization rather than confirm direct binding or functional relevance under physiological conditions.

### Cell culture and treatment

Human dermal fibroblasts (Hs 865.Sk, COBIOER Nanjing, China) were cultured in CBP61566M medium (COBIOER Nanjing, China) supplemented with 10% fetal bovine serum (FBS), 1% penicillin/streptomycin, and maintained in a humidified incubator at 37 °C with 5% CO_2_. Cells were used for experiments at passages 4–8.

For paclitaxel treatment, Hs 865.Sk were treated with concentrations of paclitaxel (10 nM and 100 nM) or vehicle control (0.1% DMSO). The treatment duration was 48 hours. Cell viability and proliferation were assessed at 24 and 48 hours post-treatment.

### Cell viability assay

Cell viability was assessed using the Cell Counting Kit-8 (CCK-8, Sigma-Aldrich). Cells were seeded in 96-well plates at a density of 2000–5000 cells per well and allowed to adhere overnight. Following paclitaxel treatment, 10 µL of CCK-8 reagent was added to each well, and the plates were incubated for 1–2 hours at 37 °C. The absorbance at 450 nm was measured using a microplate reader (Thermo Fisher). The cell viability was calculated as the percentage of the control group (vehicle-treated cells). For each concentration, experiments were performed with 3 independent biological replicates, each measured with 3 technical replicates. Statistical significance between groups was assessed using the t-test, and error bars in graphs represent standard deviation (SD).

### Quantitative PCR analysis

Total RNA was extracted using TRIzol reagent (Thermo Fisher) according to the manufacturer’s protocol. RNA concentration and quality were assessed using a NanoDrop spectrophotometer (Thermo Fisher). cDNA was synthesized using the High-Capacity cDNA Reverse Transcription Kit (Thermo Fisher) according to the manufacturer’s instructions.

The qPCR analysis was performed using SYBR Green PCR Master Mix (Thermo Fisher) on a StepOnePlus Real-Time PCR System (Applied Biosystems). The following gene-specific primers were used. AKT1: Forward: 5’-GTCATCGAACGCACCTTCCAT-3’, Reverse: 5’-AGCTTCAGGTACTCAAACTCGT-3’; BCL2: Forward: 5’-ATCGCCCTGTGGATGACTGAGT-3’, Reverse: 5’-GCCAGGAGAAATCAAACAGAGGC-3’; BAX: Forward: 5’-CCCGAGAGGTCTTTTTCCGAG-3’, Reverse: 5’-CCAGCCCATGATGGTTCTGAT-3’; COL1A1: Forward: 5’- ATCAACCGGAGGAATTTCCGT-3’, Reverse: 5’-CACCAGGACGACCAGGTTTTC-3’; ACTA2 (α-SMA): Forward: 5’-CTATGAGGGCTATGCCTTGCC-3’, Reverse: 5’-GCTCAGCAGTAGTAACGAAGGA-3’; TGF-β: Forward: 5’-CAATTCCTGGCGATACCTCAG-3’, Reverse: 5’-GCACAACTCCGGTGACATCAA-3’; GAPDH (housekeeping gene): Forward: 5’- GTCTCCTCTGACTTCAACAGCG-3’, Reverse: 5’-ACCACCCTGTTGCTGTAGCCAA-3’.

The PCR conditions were as follows: 95 °C for 10 minutes for initial denaturation, followed by 40 cycles of 95 °C for 15 seconds, 60 °C for 1 minute. Each qPCR reaction was run in 3 technical replicates. Data were analyzed using the ^ΔΔCt^ method, and results were normalized to GAPDH expression. Statistical significance was evaluated using the t-test for pairwise comparisons, with error bars representing SD.

## Results

### FAERS-based pharmacovigilance analysis

From Q1–2004 to Q4 2024, a total of 89 AE reports related to SSc following paclitaxel exposure were identified in the FAERS database. Among these, three preferred terms (PTs)—SCLERODERMA, SCLERODERMA-LIKE REACTION, and SYSTEMIC SCLERODERMA - met the threshold for inclusion (n≥3). Reports with SCLERODERMA OVERLAP SYNDROME and CREST SYNDROME were excluded due to insufficient frequency.

Descriptive analysis (**Table 1**) showed that the majority of reported systemic sclerosis-related adverse events occurred in female patients (75.3%), with the highest reporting rate observed in the 45–64 age group (44.9%). Most reports originated from Europe (46.1%) and Asia (44.9%). Regarding reporter identity, healthcare professionals—particularly other health experts (62.9%) and physicians (19.1%)—were the primary reporters. Hospitalization (22.5%) and other medically important events (59.6%) were the most frequently reported clinical outcomes. Body weight was reported in 11 out of 89 cases (12.3%), and due to the high rate of missing values, it is presented here for descriptive purposes only. No formal subgroup analysis based on body weight was performed.

**Table 1:** Descriptive statistics of adverse event reports related to paclitaxel-associated systemic sclerosis.

Item	Level	Count (%)
N		89
Gender	Female	67 (75.3)
	Male	11 (12.4)
	Unknown	11 (12.4)
Age	18-44	5 (5.6)
	45-64	40 (44.9)
	≥65	27 (30.3)
	NS	17 (19.1)
Weight	<80 kg	9 (10.1)
	≥80 kg	2 (2.2)
	Unknown	78 (87.6)
Occupation of the reporter	Registered nurse	7 (7.9)
	Medical doctor	17 (19.1)
	Pharmacist	3 (3.4)
	Other healthcare professionals	56 (62.9)
	Unknown	6 (6.7)
Continent of the reporter’s country	Asia	40 (44.9)
	Europe	41 (46.1)
	North America	3 (3.4)
	South America	3 (3.4)
	Others	2 (2.2)
Outcome	Death	7 (7.9)
	Disability	8 (9.0)
	Hospitalization	20 (22.5)
	Other outcome	53 (59.6)
	Unknown	1 (1.1)

**Figure 2:**
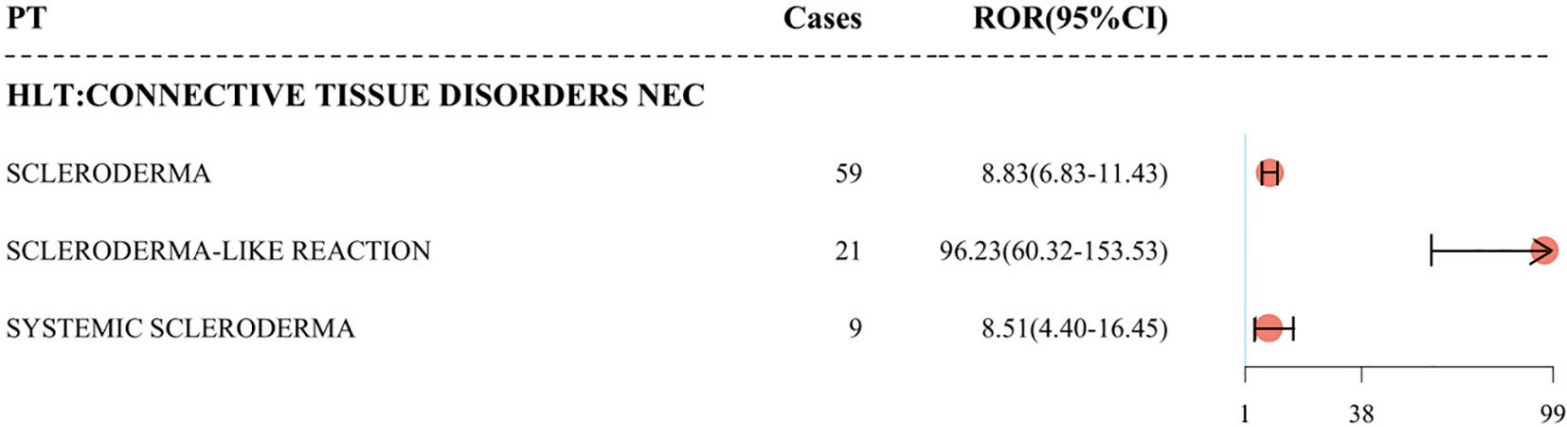
Forest plot of disproportionality analysis for paclitaxel-associated systemic sclerosis adverse events. Error bars represent 95% confidence intervals (CI).

**Figure 3:**
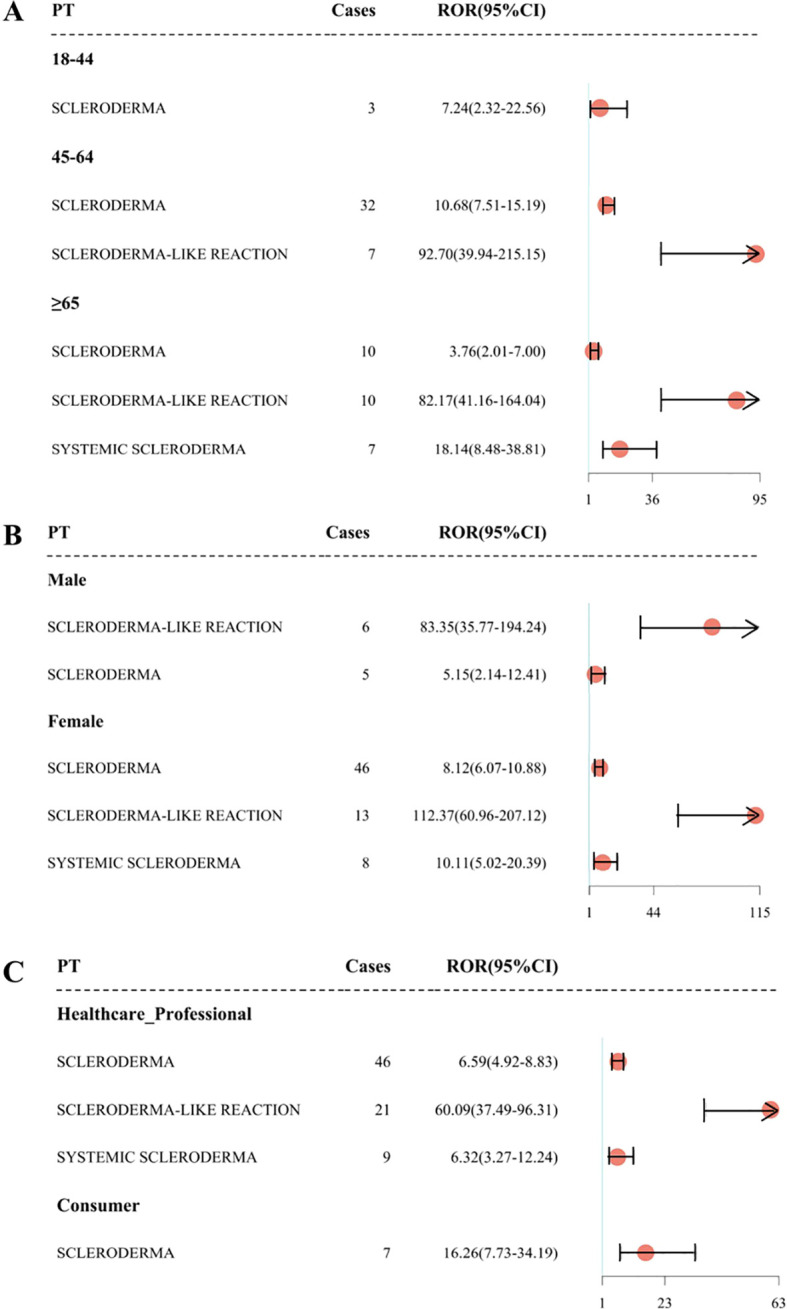
Subgroup analysis of disproportionality signals for paclitaxel-associated systemic sclerosis. The forest plots display the reporting odds ratios (RORs) and 95% confidence intervals across different subgroups: **(A)** age, **(B)** sex, and **(C)** reporter occupation. All subgroups showed positive signals (n ≥ 3, ROR > 1), indicating a consistent association between paclitaxel and systemic sclerosis across demographic and regional strata. Error bars represent 95% confidence intervals (CI).

Disproportionality analysis revealed statistically significant signals for all three PTs (**Figure 2**). For SCLERODERMA: n = 59, ROR = 8.83, PRR = 6.83, χ² = 402.52, IC025 = 2.74. For SCLERODERMA-LIKE REACTION: n = 21, ROR = 96.23, PRR = 96.21, χ² = 1659.03, IC025 = 5.67. For SYSTEMIC SCLERODERMA: n = 9, ROR = 8.51, PRR = 6.83, χ² = 58.67, IC025 = 2.15. Subgroup analysis by sex, age, and region confirmed consistent signals across multiple strata (**Figure 3**), indicating a robust association between paclitaxel and SSc-related AE. The occurrence of SCLERODERMA-related AEs was primarily concentrated in individuals aged 45–64 years (ROR = 10.68), and predominantly observed in female patients (ROR = 8.12). Although the absolute number of reports for individual PTs, particularly SYSTEMIC SCLERODERMA, was limited, paclitaxel-associated signals were rare but consistently observed across multiple clinically related PTs, supporting their relevance as hypothesis-generating safety signals rather than definitive epidemiological evidence. These findings suggest that paclitaxel is associated with SSc-related adverse event signals, with potential variation across sex and age subgroups. These findings are exploratory and hypothesis-generating, rather than definitive evidence of epidemiological causality.

### Mendelian randomization analysis

**Figure 4:**
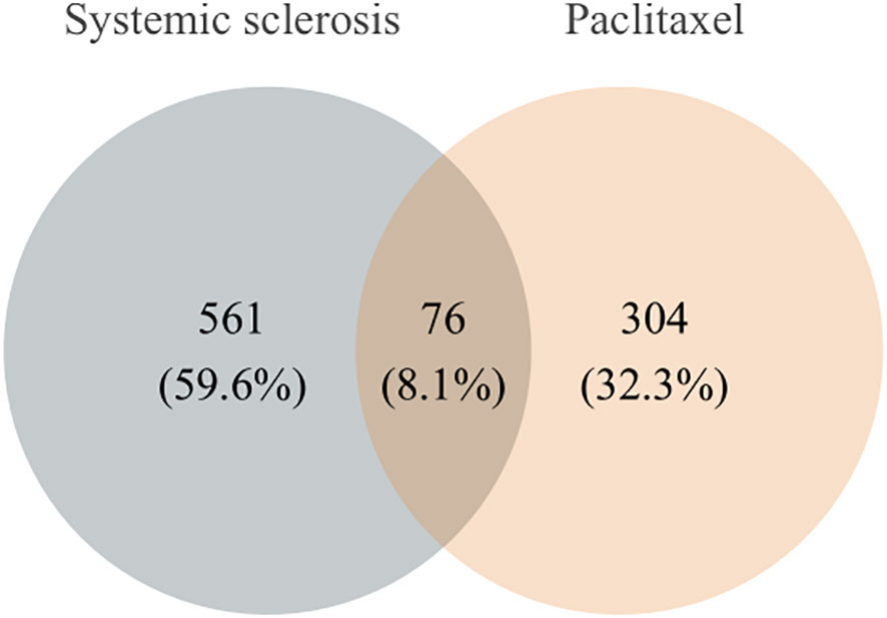
Venn diagram of shared molecular targets between paclitaxel and systemic sclerosis. Paclitaxel-associated targets (n = 380) were identified using CTD, STITCH, and SwissTargetPrediction databases. Systemic sclerosis-related genes (n = 637) were retrieved from GeneCards and DisGeNET. A total of 76 overlapping genes were identified as potential shared targets involved in paclitaxel-associated systemic sclerosis.

A total of 380 paclitaxel-related targets were identified from CTD, STITCH, and SwissTargetPrediction databases. Concurrently, 637 systemic sclerosis-associated genes were collected from GeneCards and DisGeNET. The intersection yielded 76 shared genes (**Figure 4**), which were considered candidate genes of paclitaxel-associated SSc.

**Figure 5:**
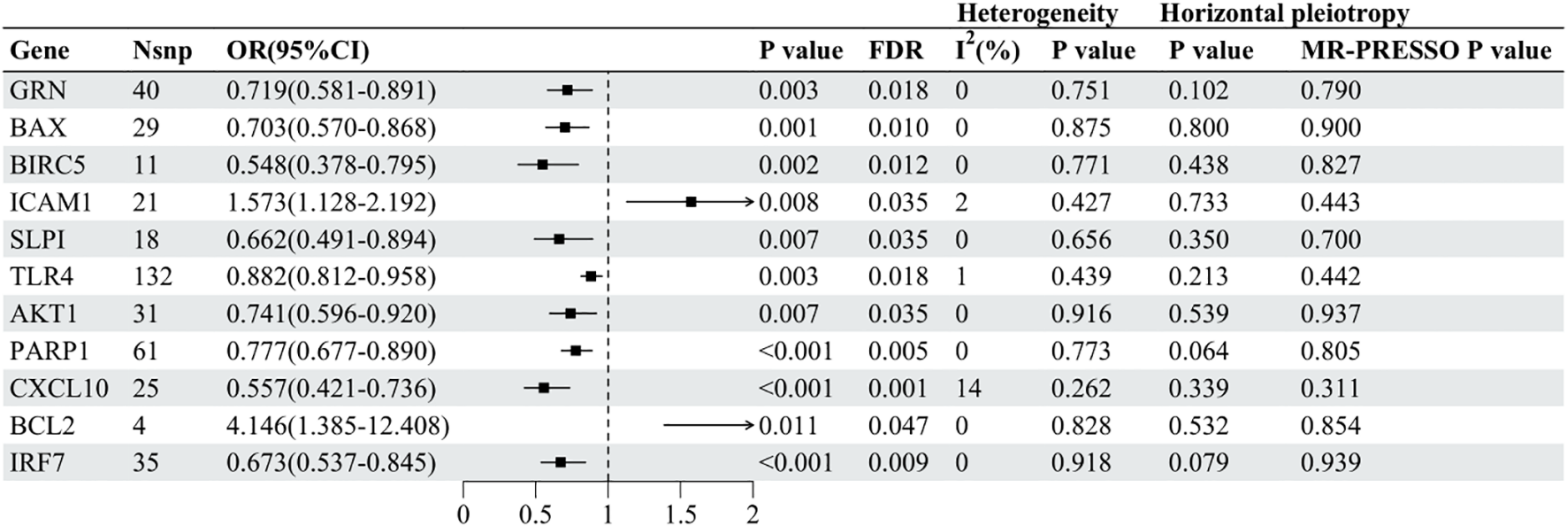
Forest plot of Mendelian randomization results for candidate targets associated with systemic sclerosis.

Of the 76 shared genes, 60 had eligible eQTLs available in the IEU OpenGWAS database. After instrument selection and quality control, 1739 SNPs were retained as valid instruments. Using IVW as the primary method, we identified 13 genes with statistically significant associations with SSc. After excluding results with heterogeneity or horizontal pleiotropy, 11 genes remained as potential candidate targets (**Figure 5**). For example, GRN was negatively associated with SSc risk (OR = 0.719, 95% CI: 0.581–0.891, P = 0.003, FDR = 0.018), with no evidence of heterogeneity (I² = 0%, P = 0.751) or pleiotropy (MR-Egger intercept P = 0.102, MR-PRESSO P = 0.790). These results are exploratory and indicate potential associations rather than confirmed causal relationships.

### Functional enrichment analysis

**Figure 6:**
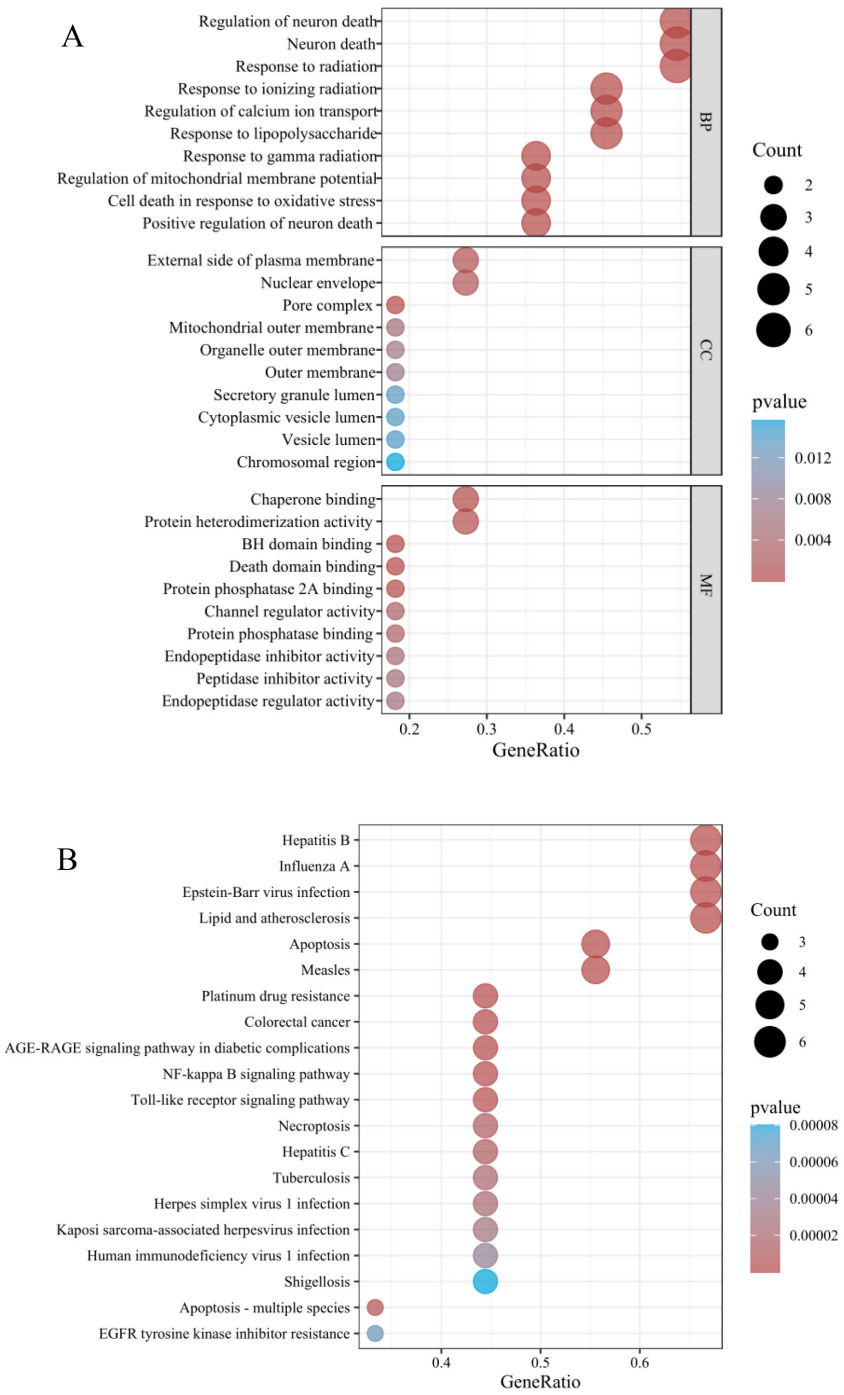
Functional analysis of 11 candidate genes associated with systemic sclerosis. **(A)** Gene Ontology (GO) enrichment analysis. **(B)** KEGG pathway enrichment analysis. P values shown are adjusted for multiple testing using the Benjamini–Hochberg method.

GO enrichment analysis of the 11 candidate genes revealed biological processes related to the regulation of neuron death and cell death in response to oxidative stress. Cellular components were enriched in pore complexes and secretory granule lumens, while molecular functions included BH domain binding and death domain binding (**Figure 6A**). KEGG analysis showed significant enrichment in pathways such as Hepatitis B, Influenza A, Epstein-Barr virus infection, lipid and atherosclerosis, and apoptosis (**Figure 6B**). Although the measles pathway showed a similar gene ratio and adjusted P value, apoptosis was highlighted due to its direct relevance to fibroblast survival, immune dysregulation, and fibrosis progression in systemic sclerosis. These results suggest that the core targets are involved in immune response, oxidative stress, and lipid metabolism, pathways closely linked to the pathophysiology of SSc. These findings are hypothesis-generating and require further experimental validation.

### Protein–protein interaction network

**Figure 7:**
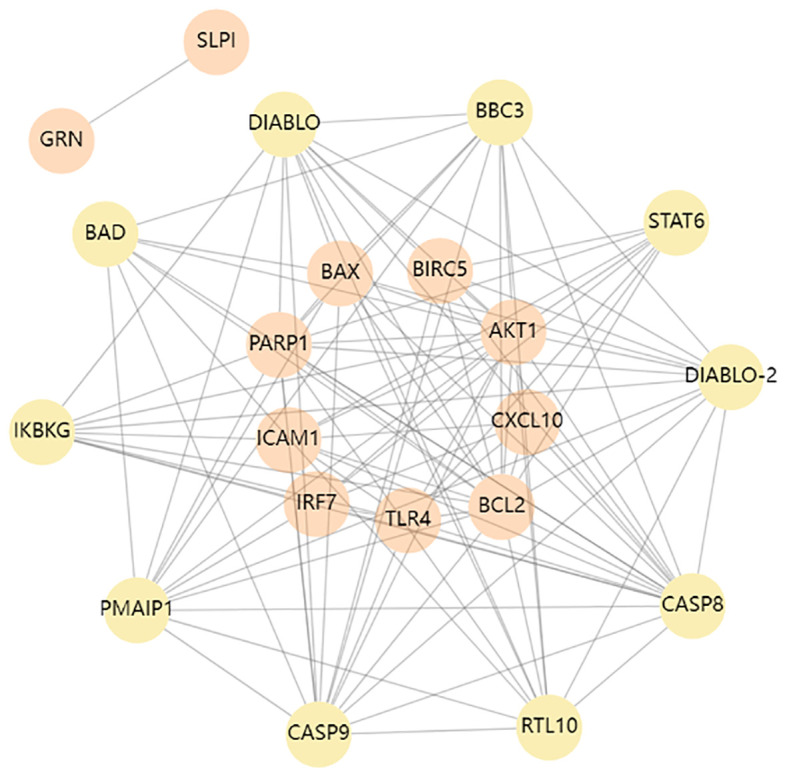
Protein-protein interaction (PPI) network of the 11 candidate genes associated with systemic sclerosis. The PPI network was constructed using the STRING database and visualized in Cytoscape. It includes 21 nodes and 108 edges, with an average node degree of 11.26. Orange nodes represent the 11 genes with significant associations from Mendelian randomization analysis, while light yellow nodes represent interacting proteins. Hub genes were identified using the Maximal Clique Centrality (MCC) algorithm.

The PPI network constructed from the 11 candidate genes comprised 21 nodes and 108 edges, with an average node degree of 11.26 (**Figure 7**). Using the Maximal Clique Centrality (MCC) algorithm, five hub genes were identified. Among them, AKT1 and BCL2 were both identified as potential associated genes in MR analysis, highlighting their status as candidate targets for further investigation (**Table 2**). These analyses provide exploratory evidence for prioritizing candidate targets.

**Table 2:** Top 5 hub genes identified from the PPI network using the Maximal Clique Centrality (MCC) algorithm.

Rank	Name	Score
1	AKT1	3671676
2	CASP8	3671406
3	BCL2	3671310
4	CASP9	3670080
5	PMAIP1	3669126

### Molecular docking

**Figure 8:**
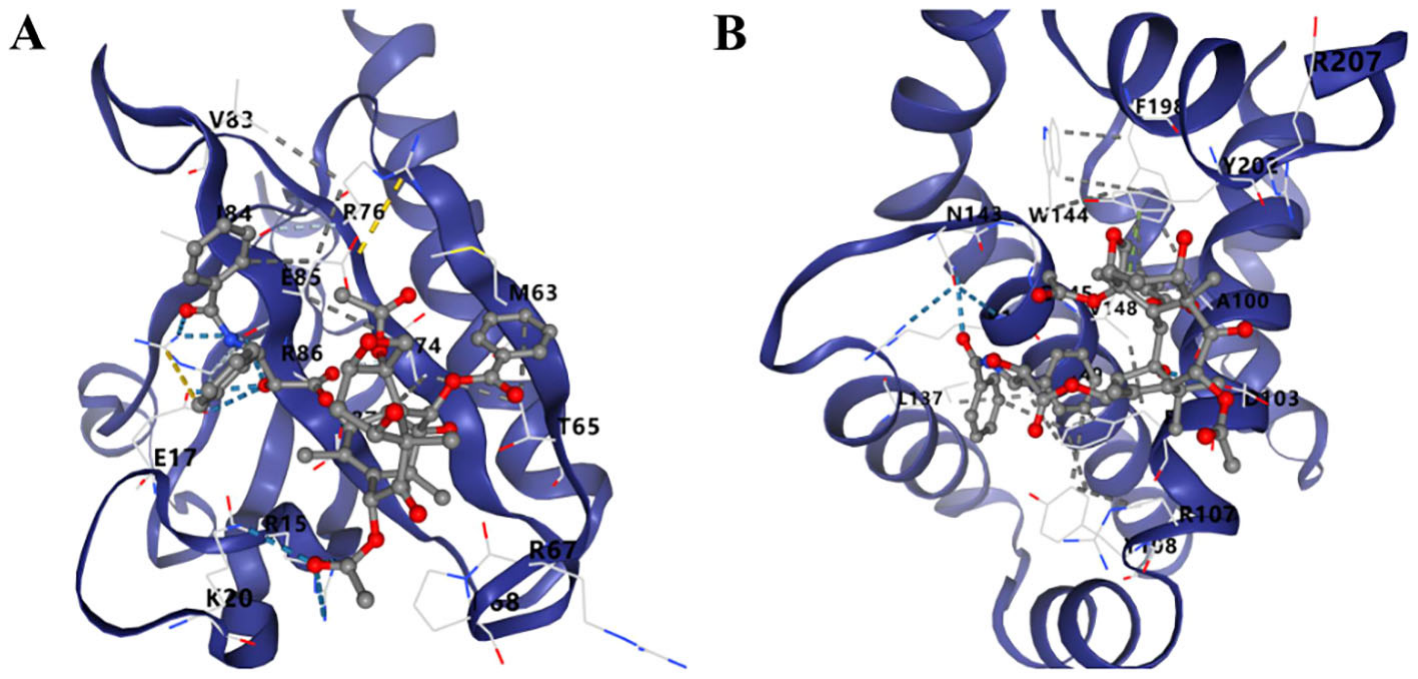
Molecular docking analysis of paclitaxel with core target proteins AKT1 and BCL2. **(A)** Paclitaxel binding to AKT1; **(B)** Paclitaxel binding to BCL2. Docking simulations were performed using CB-Dock2 and AutoDock Vina.

Docking simulations using CB-Dock2 produced raw Vina scores below -5 kcal/mol for both AKT1 (-6.5 kcal/mol) and BCL2 (-7.1 kcal/mol) (**Figures 8A, B**), consistent with precedent in a comparable exploratory docking study (31). Key residues involved in the predicted interactions are located within functional domains reported in UniProt. For AKT1 (**Figure 8A**), residues R15, E17, K20, M63, T65, P68, R76, V83, I84, E85, and R86 were predicted to form hydrogen bonds, electrostatic, or van der Waals contacts with paclitaxel. Several of these residues are located within or adjacent to the pleckstrin homology (PH) domain, which is involved in membrane recruitment and AKT activation. For BCL2 (**Figure 8B**), residues R98, A100, D103, R107, L137, N143, W144, G145, V148, F198, and Y202 were predicted to participate in similar interactions. Several predicted interacting residues were located within or near regions associated with the BH domains and the hydrophobic groove involved in apoptotic regulation. These interactions are based on computational docking predictions and are exploratory. Further structural and biochemical validation is warranted.

### *In vitro* supportive evidence for candidate targets identified in paclitaxel-associated SSc

**Figure 9:**
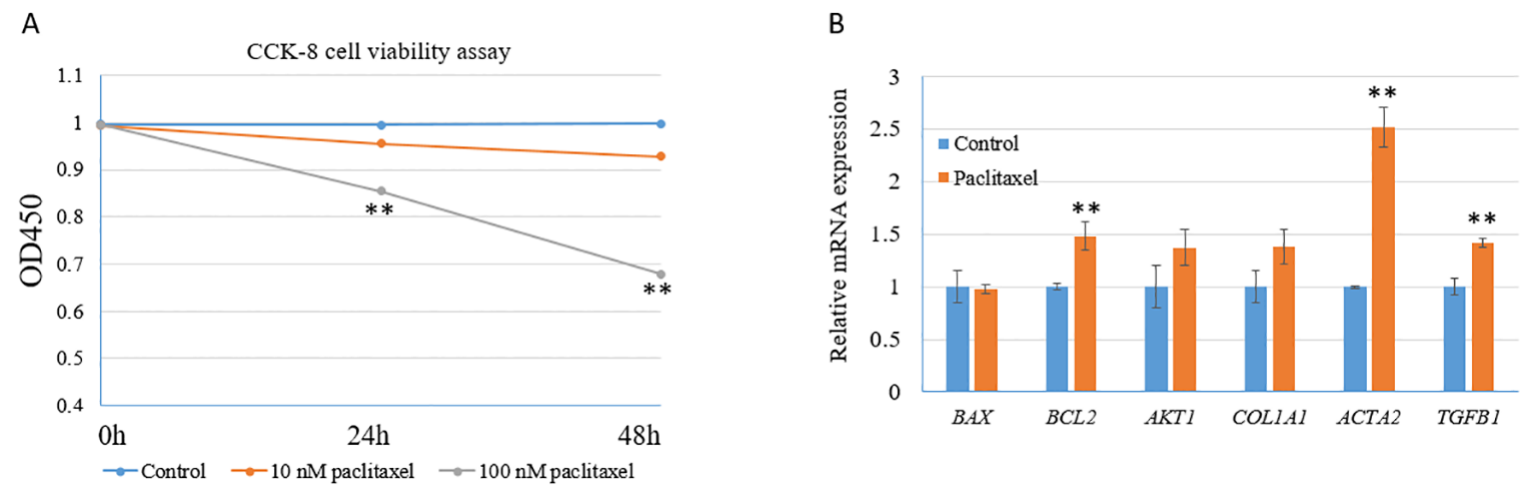
Exploratory evaluation of candidate genes in Hs 865.Sk fibroblasts. **(A)** Dose-response curve for paclitaxel treatment in HDFs as measured by CCK-8 assay (n = 3 biological replicates, each with 3 technical replicates). **(B)** Gene expression analysis of BAX, BCL2, AKT1, COL1A1, ACTA2, and TGF-β in Hs 865.Sk treated with 10 nM paclitaxel (n = 3 biological replicates, with 3 technical replicates; error bars = SD). **P < 0.01, t-test.

In human dermal fibroblasts (Hs 865.Sk), paclitaxel at 10 nM did not significantly reduce cell viability after 48 h (**Figure 9A**, P>0.05), while 100 nM caused significant cytotoxicity (P<0.01). The 10 nM concentration was considered sub-toxic based on CCK-8 results showing no significant reduction in cell viability compared with control, consistent with concentrations commonly used to assess non-cytotoxic signaling effects. qPCR analysis revealed that 10 nM paclitaxel induced a 1.42-fold increase in TGF-β and a 1.48-fold increase in BCL2 expression relative to vehicle (P<0.01), accompanied by a 2.52-fold increase in ACTA2 (P<0.01). The expression of BAX and BCL2 showed opposite trends after paclitaxel treatment, resulting in a decreased BAX/BCL2 ratio, suggesting a shift toward an anti-apoptotic profile (**Figure 9B**). These *in vitro* findings provide preliminary supportive biological evidence that sub-toxic paclitaxel exposure may be associated with fibroblast activation, in line with the pathways highlighted by the MR and network analyses. Further studies, including protein-level validation and functional perturbation in animal models, are required to confirm mechanistic roles.

## Discussion

Paclitaxel, a widely used chemotherapeutic agent, has increasingly been implicated in drug-related SSc, a rare and complex autoimmune disease characterized by widespread fibrosis and vascular dysfunction (32). In this study, by combining multiple toxicogenomic databases and disease gene repositories, we identified 76 shared targets between paclitaxel and SSc, 60 of which were further tested using MR. MR analyses suggested that 11 genes were potentially associated with SSc susceptibility, supported by statistical sensitivity analyses. Functional enrichment analyses indicated that these genes were involved in pathways such as oxidative stress response, regulation of cell death, lipid metabolism, and apoptosis—all of which are relevant to SSc pathophysiology (33). Of particular note, AKT1 and BCL2 emerged as central nodes within the PPI network, highlighting their potential relevance as candidate targets for further investigation. Molecular docking simulations were performed to explore the potential binding of paclitaxel to these proteins. Collectively, these findings offer hypothesis-generating insights into pathways that may be relevant to fibrotic and immune dysregulation in SSc.

The association between paclitaxel and systemic sclerosis has previously been reported in both clinical and experimental studies, albeit in a limited scope. Several case reports and pharmacovigilance analyses have documented scleroderma-like syndromes in patients receiving paclitaxel-based chemotherapy, including skin thickening, Raynaud’s phenomenon, digital ulcers, and pulmonary fibrosis—features highly reminiscent of SSc. In a cohort analysis, paclitaxel was among several agents associated with the emergence of connective tissue disease symptoms following cancer therapy (10). Moreover, pathological changes observed in paclitaxel-related fibrotic lesions in animal models—such as increased collagen deposition and fibroblast activation—closely mirror the histological hallmarks of SSc (34). These findings suggest that paclitaxel may potentially contribute to SSc-like manifestations in susceptible individuals. However, the molecular mechanisms underlying this phenomenon remain largely unclear and require further elucidation.

Apoptosis dysregulation has long been implicated in the pathogenesis of SSc, particularly concerning endothelial injury, fibroblast persistence, and immune cell imbalance. Both BCL2 and AKT1 are key regulators of apoptosis and cell survival pathways and have been independently linked to SSc progression (35). BCL2, a classic anti-apoptotic protein, is overexpressed in dermal fibroblasts and infiltrating lymphocytes in SSc lesions, contributing to their resistance to apoptosis and promoting chronic fibrosis (35–37). Similarly, AKT1, as a central node in the PI3K/AKT signaling axis, regulates fibroblast proliferation and myofibroblast differentiation; its aberrant activation has been reported in both skin and lung tissues of SSc patients (35). In the current study, AKT1 and BCL2 were highlighted as potential targets involved in paclitaxel-associated pathways, providing preliminary, hypothesis-generating evidence that apoptotic resistance and survival signaling may be relevant to drug-associated fibrotic responses. Further experimental validation is required to confirm their mechanistic roles.

In our study, paclitaxel treatment significantly increased TGF-β1, ACTA2, and BCL2 mRNA levels, providing preliminary evidence of potential associations with pro-fibrotic gene expression patterns and fibroblast survival signaling. In contrast, AKT1 expression showed no significant change, suggesting that paclitaxel may influence AKT signaling at the post-translational level (e.g., phosphorylation) rather than through transcriptional regulation. These findings are exploratory and hypothesis-generating, and are broadly consistent with previous studies showing that TGF-β1 acts upstream of the PI3K/AKT pathway to influence fibroblast activation and extracellular matrix deposition in SSc (38).

Several limitations of our study must be acknowledged. First, the FAERS data rely on spontaneous reports, which may introduce reporting bias or underreporting (39). Future studies integrating VigiBase or EudraVigilance data are warranted to further validate these signals. Second, our MR analyses were confined to populations of European descent, and thus, findings may not generalize across diverse populations. Therefore, the observed associations may not be directly generalizable to other ethnic groups, in whom the incidence and genetic architecture of SSc differ substantially. Third, only two paclitaxel concentrations were tested, and future studies using a broader dose range and additional functional assays are needed to further validate dose-dependent effects. Although AKT1 and BCL2 were prioritized based on network analysis and mRNA-level changes, we acknowledge that protein-level validation (e.g., phospho-AKT1, BCL2) is lacking, and the proposed TGF-β/PI3K/AKT pathway model remains a hypothesis requiring experimental confirmation. Further functional validation using siRNA-mediated knockdown, rescue experiments, and extracellular matrix deposition assays is required. These considerations underscore that our findings are hypothesis-generating and exploratory rather than confirming definitive mechanisms.

In conclusion, we identified a significant association between paclitaxel and SSc through pharmacovigilance analysis, and AKT1 and BCL2 were highlighted as exploratory candidate targets for prioritization in future studies. This integrative, hypothesis-generating approach provides a framework for exploring drug-associated autoimmunity and may inform future studies aimed at early risk identification and therapeutic strategies in susceptible populations.

## Data Availability

Publicly available datasets were analyzed in this study. The dataset analyzed in the study is available at the FAERS, CTD, DisGeNET, OpenGWAS, and STRING databases.
